# Transcriptome of *Kurthia gibsonii* TYL-A1 Revealed the Biotransformation Mechanism of Tylosin

**DOI:** 10.3390/microorganisms12122597

**Published:** 2024-12-16

**Authors:** Ye Wang, Cuizhu Zhao, Boyu Zhao, Xinran Duan, Peng Hao, Xiaojun Liang, Lianyu Yang, Yunhang Gao

**Affiliations:** 1 College of Veterinary Medicine, Jilin Agricultural University, Changchun 130118, China; 18904404595@163.com (Y.W.); zhaocuizhu2024@163.com (C.Z.); zhaobooyu@163.com (B.Z.); 15048180317@163.com (X.D.); hhaopengg@163.com (P.H.); 2 Institute of Animal Science, Ningxia Academy of Agriculture and Forestry Sciences, Yinchuan 750002, China; lxj0520@163.com

**Keywords:** tylosin, antioxidant reaction, degradation, transcriptome sequencing

## Abstract

Tylosin (TYL) pollution has aroused widespread concern, and its existence poses a serious threat to the environment and human health. Microbial degradation of antibiotics is considered to be an effective strategy to reduce the environmental impact of antibiotics, but its degradation mechanism is still unclear. In this study, transcriptome analysis was combined to explore the response mechanism of *K. gibsonii* strain TYL-A1 under TYL stress. The results showed that the strain showed a significant antioxidant response under TYL stress to cope with TYL-induced cell damage. TYL also increased the level of intracellular reactive oxygen species (ROS), damaged the integrity of the cell membrane, and inhibited the growth of strain TYL-A1. Transcriptome sequencing showed that under TYL exposure conditions, 1650 DEGs in strain TYL-A1 showed expression changes, of which 806 genes were significantly up-regulated and 844 genes were significantly down-regulated. Differentially expressed DEGs were significantly enriched in pathways related to metabolism, biosynthesis, and stress response, and tricarboxylic acid cycle, oxidative phosphorylation, and carbon metabolism genes were significantly up-regulated. In conclusion, this study provides novel insights regarding the degradation of TYL by *K. gibsonii* TYL-A1.

## 1. Introduction

Human health and ecosystem integrity are seriously threatened by misuse and overuse of antibiotics in livestock and poultry farming [1]. A wide variety of antibiotics are frequently used to treat illness or promote the growth of livestock [2,3]. However, the majority of antibiotics do not undergo complete metabolism but are excreted in animal feces and urine as the native compound orvarious metabolites and chemicals [4,5]. Therefore, when animal waste is dispersed in the environment (e.g., applied to farmland), it often has residual biological toxicity and promotes the development of antibiotic-resistant bacteria (ARB), which can cause environmental pollution [6,7]. TYL, a macrolide antibiotic produced by *Streptomyces floridus*, is widely used in livestock and poultry production and for the treatment of animal diseases or the promotion of growth, due to its good pharmacokinetic and growth-promoting properties [8,9]. However, residual TYL in livestock waste can spread to soil and water, leading to detrimental environmental effects [10]. The impact and potential risks associated with TYL residues are an impetus to identify efficient ways to remove TYL from the environment.

There are several means to get rid of or degrade antibiotics, including physical adsorption, advanced oxidation, and biodegradation; the latter is preferred due to its great effectiveness, affordability, controllability, and environmental friendliness [11,12]. The basis of microbial degradation is antibiotic-resistant microorganisms, which, when exposed to antibiotics, produce antibiotic-degrading enzymes [13].

Degradation of organic compounds by bacterial strains is mainly due to intracellular enzymes, including adsorption, transmembrane transport, and enzymatic reactions [14,15]. First, microorganisms adapt to changes in the external environment, e.g., the presence of antibiotics, by regulating their physiological and biochemical processes to facilitate the transport and metabolism of the antibiotic. Transmembrane transport has a crucial role in the biodegradation of petroleum hydrocarbons, suggesting that the initial stage of biodegradation involves the transfer of hydrophobic substrates across the cell membrane [16]. To minimize damage due to environmental pollutants, bacteria can activate antioxidant systems and modify their metabolism, protein synthesis, and gene expression [17].

Microorganisms degrade organic pollutants and concurrently, pollutants act on the microorganisms; this reciprocal relationship causes pollutants to have certain effects on microorganisms, including responses to unfavorable conditions. Most current studies on antibiotic biodegradation focus on isolating, identifying and characterizing the degrading bacteria. For example, *Providencia stuartii* TYL-Y13 completely degraded 100 mg/L of TYL within 15 h [18] whereas *Klebsiella oxytoca* TYL-T1 caused 99.34% degradation of 25 mg/L of TYL [19]. However, intracellular antioxidant responses and degradation mechanisms are not well characterized. Studies have shown that *Kurthia* has a variety of biological functions and can degrade a variety of organic pollutants, which may be related to its production of microbial surfactants [20]. In addition, *Kurthia* also has the advantages of salt, alkali [21], and organic solvent resistance. In this study, cell membrane hydrophobicity, cell membrane permeability, and some antioxidant indexes of strain TYL-A1 stimulated by various concentrations of TYL were investigated. These approaches and transcriptome sequencing were used to elucidate the mechanism of how pollutants affect microorganisms and in particular, to elucidate degradation of TYL by *K.gibsonii* TYL-A1.

## 2. Materials and Methods

### 2.1. Chemicals and Media

TYL-A1 is a drug-resistant bacterium isolated and preserved by our research group and cultured using TSB medium [22].TSB is from Hope Bio-Technology Co., Ltd. (Qingdao, China). The TYL (purity ≥ 93%) was purchased from Yuanye Biotechnology Company (Shanghai, China), methanol and acetonitrile of HPLC grade were purchased from Fisher Scientific Co. (Shanghai, China). The formic acid (purity 95.5%) was purchased from FUCHEN Chemistry (Tianjin, China). Minimal Salt Medium (MSM; g/L) was made up of MgSO_4_-H_2_O 0.20 g, KH_2_PO_4_ 0.5 g, K_2_HPO_4_ 1.5 g, NaCl 1.0 g, and yeast extract 1.0 g. The recombinant bacteria were cultured using Luria–Bertani medium (LB; g/L), made up of tryptone 10.0 g, NaCl 10.0 g, and yeast extract 5.0 g. All other chemicals were analytical grade.

### 2.2. The Changes of Cell Membrane of Strain TYL-A1 Under TYL Exposure

#### 2.2.1. Determination of Scanning Electron Microscope (SEM) and Fourier Transform Infrared Spectrometer (FTIR)

Strain TYL-A1 was inoculated into fresh TSB to prepare a bacterial suspension, and then the pre-cultured TYL-A1 was centrifuged at 8500 rpm for 15 min at 4 °C, washed three times with sterile PBS, re-suspended, adjusted to OD_600_ = 2.0 and added to MSM for subsequent experiments. To explore effects of TYL on bacterial morphology and functional group changes, the bacterial solution was added to MSM media that included various concentrations of TYL (0, 75 and 150 mg/L) and culture for 24 h at 30 °C and 130 rpm in a dark shaker. Bacterial cells were collected, fixed with glutaraldehyde, dehydrated with anhydrous ethanol gradient, and evaluated with SEM and FTIR.

#### 2.2.2. Determination of Cell-Surface Hydrophobicity (CSH)

To investigate effects of TYL on cellular properties of strain TYL-A1 during degradation, CSH was determined using the bacterial adhesion to hydrocarbons (MATH) method. Suspensions were inoculated (2% inoculation rate) into MSM and cultured overnight in a constant temperature (30 °C) oscillating incubator at 130 rpm. The suspension of bacteria was adjusted to a cell concentration of OD_600_ = 0.6. Then, 4 mL of bacterial suspension was added to the test tube, plus 2 mL of xylene. No xylene was added to the control group. It was violently shaken at room temperature for 2 min, stationary culture for 20 min until stratified, and the lower-phase aqueous solution was quickly absorbed. The OD value was determined at 600 nm with PBS buffer as the blank control. Each process was repeated three times. Bacterial cell surface hydrophobicity (CSH%) was calculated as follows:

CSH% = (OD_control group_ − OD_experimental group_)/OD_control group_ × 100%

#### 2.2.3. Determination of Membrane Permeability

Effect of TYL on membrane permeability of strain TYL-A1 during degradation was determined by the crystal violet method. For this, 2 mL of bacterial suspension was seeded into MSM with varying concentrations (25, 50, 75, 100 and 125 mg/L) of TYL and cultured in a shaker at 30 °C and 130 rpm. Cells were sampled for 24 h and then harvested and suspended in a PBS solution containing 0.1 mg/mL crystal violet. The sample was incubated at 30 °C for 10 min, centrifuged at 12,000 rpm for 20 min, and the supernatant’s OD value was determined at 590 nm. The OD value of the initial 0.1 mg/mL crystal violet solution was regarded as 100%. Percentage of crystal violet absorption was determined as follows:

Crystal violet absorption (%) = (OD_control group_ − OD _experimental group_)/OD_control group_ × 100%

### 2.3. Determination of ROS Level

First, 2 mL of bacterial solution was seeded into MSM with varying concentrations (25, 50, 75, 100 and 125 mg/L) of TYL and cultured in a shaker at 30 °C and 130 rpm. Bacterial cells treated with various concentrations (25, 50, 75, 100 and 125 mg/L) of TYL for 24 h were collected, resuspended in 2,7-dichlorofuorescein diacetate (DCFH-DA), the bacterial concentration adjusted to 1 × 10^7^ CFU, incubated at 37 °C for 20 min, mix upside down 4 times during incubation. Then, to get rid of DCFH-DA that did not enter the cells, the bacterial cells were then rinsed three times with PBS. The test sample was then detected using a fluorescence microplate reader at an excitation wavelength of 488 nm and an emission wavelength of 525 nm.

### 2.4. Determination of Antioxidant Reaction Metrics

Degrading bacteria cells stressed with various concentrations (25, 50, 75, 100 and 125 mg/L) of TYL for 24 h were collected and assessed based on the kit instructions of Nanjing Jiengcheng Bioengineering Research Institute. Superoxide dismutase (SOD), catalase (CAT), and malondialdehyde (MDA) were measured to assess the antioxidant reaction of bacteria while degrading TYL.

### 2.5. Determination of ATP Content

Bacterial cells exposed to various concentrations (0, 25, 50, 75,100 and 125 mg/L) of TYL for 24 h were collected, washed 3 times with sterile PBS, and then lysed by ultrasound. The supernatant was collected following 5 min of centrifugation at 12,000× *g*, and the Nanjing Jiancheng Bioengineering Institute manufacturer’s instructions were followed in determining the ATP concentrations.

### 2.6. Transcriptome Sequencing

Easy Pure RNA Kit (TransGen, Biotechnology, Beijing, China) was used to extract total RNA from CK and TG groups, and three biological replicates were performed. The second-strand cDNA was produced by reverse transcription of RNA into the first-strand cDNA using arbitrary primers. A QiaQuick PCR extraction kit (Agilent Technologies, Santa Clara, CA, USA) was used to purify the cDNA synthesis, which was then added to PolyA after being finalized and corrected. An Illumina HiSeq2500 (NEB, Ipswich, MA, USA) was used for sequencing and polymerase chain reaction amplification (PCR), and transcriptome data were assembled and annotated. TG (experimental group) and CK (control group) cDNA libraries were assembled, sequenced, and assessed. Beijing, China-based Nuohe Zhiyuan Technology Co., Ltd. executed the aforementioned procedures. Following identification of genes that were differentially expressed (DEGs) during TYL stress using padj < 0.05 and |log2Foldchange| > 0, pathway enrichment analysis was conducted using DEGs from Gene Ontology (GO) and Genes and Genomes (KEGG) (Accession Number PRJNA1164894).

### 2.7. Validation of Transcriptome Sequencing Data

RT-qPCR was used to confirm the transcriptome sequencing data’s accuracy. Strain TYL-A1 specific primers were designed with the aid of SnapGene (6.0.2) software and the RT-qPCR reaction was performed as described in [23]. The method was calibrated using 16 S rRNA expression.

### 2.8. Expression of the Degradation Gene Heterologously

Through GO and KEGG annotation, Four potential degradation genes (GM002829, GM002830, GM000690 and GM001961) were screened and heterologously expressed. These genes are hydrolase genes and phosphotransferase genes that may play a role in the degradation of TYL, and are up-regulated. TYL-A1 genes were cloned into plasmid pET-32a after being amplified from genomic DNA. For heterologous expression, the recombinant plasmids were introduced into *E. coli* BL21 (DE3) cells. Isopropyl β-D-thiogalactopyranoside (IPTG) induced recombinant bacterial cells were gathered and inoculated in MSM medium with 25 mg/L TYL. Cultured for 24 h at 30 °C, *E. coli* BL21 (DE3) cells containing empty pET-32a vector were used as control. In order to determine whether the gene is a key gene for the degradation of TYL, we used HPLC to detect TYL. The method for evaluating TYL residues was based on our previous studies [22].

## 3. Results

### 3.1. Cell Membrane Reaction Under TYL Exposure

**Figure 1:**
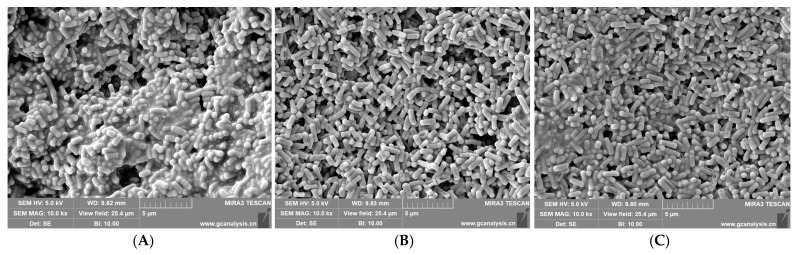
SEM strain under TYL stress: (**A**) 0 mg/L; (**B**) 75 mg/L; and (**C**) 150 mg/L.

To investigate the impacts of various concentrations of TYL on the degrading bacteria TYL-A1, bacterial surface morphology was examined with SEM (Figure 1). Bacteria that grew for 24 h in the MSM medium without TYL were full, smooth, and uniform in size. However, as TYL concentrations increased, bacterial cells had varying degrees of adhesion and depression.

**Figure 2:**
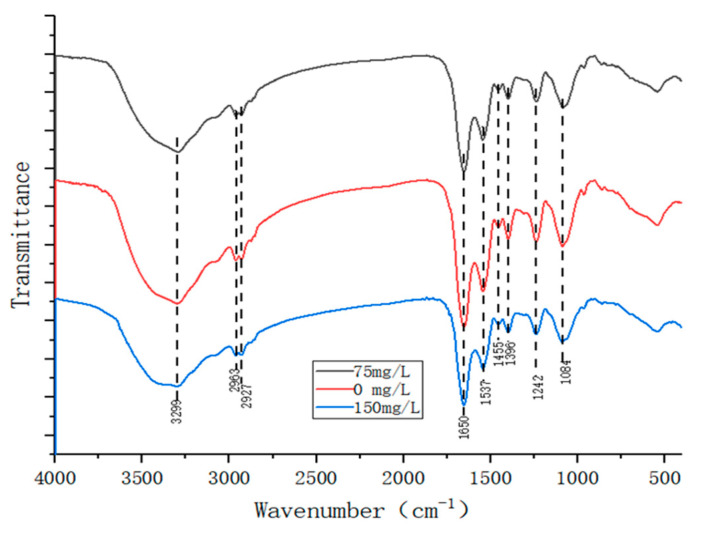
FTIR of strain TYL-A1 in response to various concentrations (0, 75 and 150 mg/L) of TYL.

From the results of FTIR measurement (Figure 2), it can be seen that in the infrared spectra of Gram-positive bacteria treated with various concentrations of TYL, the broad peak with a wave number of 3299 cm^−1^ was the hydroxyl-OH stretching vibration peak, whereas double peaks at 2963 and 2927 cm^−1^ were attributed to the C-H antisymmetric stretching vibration and symmetric stretching vibration peaks, respectively, in the saturated hydrocarbon methyl group. The 1650 cm^−1^ peak was the C=C stretching vibration peak, the 1455 and 1396 cm^−1^ peaks were the C-H bending vibration peaks in the saturated hydrocarbon group, and the 1242 cm^−1^ peak was the C-N stretching vibration peak. The 1084 cm^−1^ peak was the C-O stretching vibration peak [24]. The physical structure of the functional groups of the strain TYL-A1 changed after treatment with TYL. In response to 150 mg/L TYL, the intensity of each characteristic peak of the strain decreased, implying a high concentration of TYL may inhibit bacterial growth.

**Figure 3:**
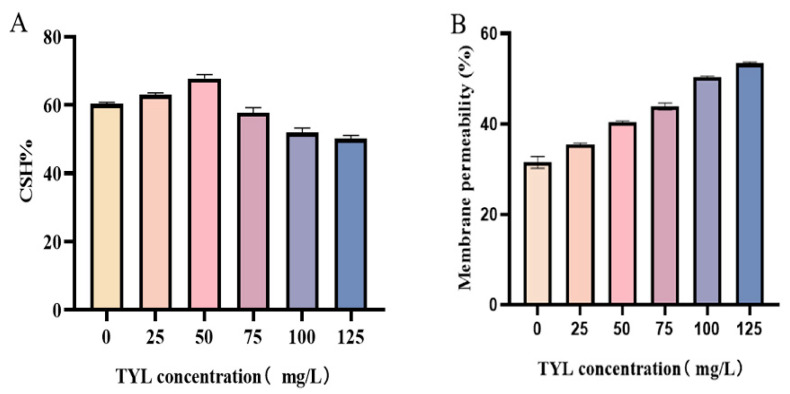
Effect of various concentrations of TYL on (**A**) CSH and (**B**) membrane permeability in strain TYL-A1.

In Figure 3A, the impact of varying TYL exposure concentrations on strain TYL-A1’s CSH was ascertained. After 24 h of reaction, with the increase in TYL concentration, the CSH change indicated a pattern of initially rising and then falling. Furthermore, as TYL concentrations increased, the membrane permeability of TYL-A1 also increased (Figure 3B).

### 3.2. ROS

**Figure 4:**
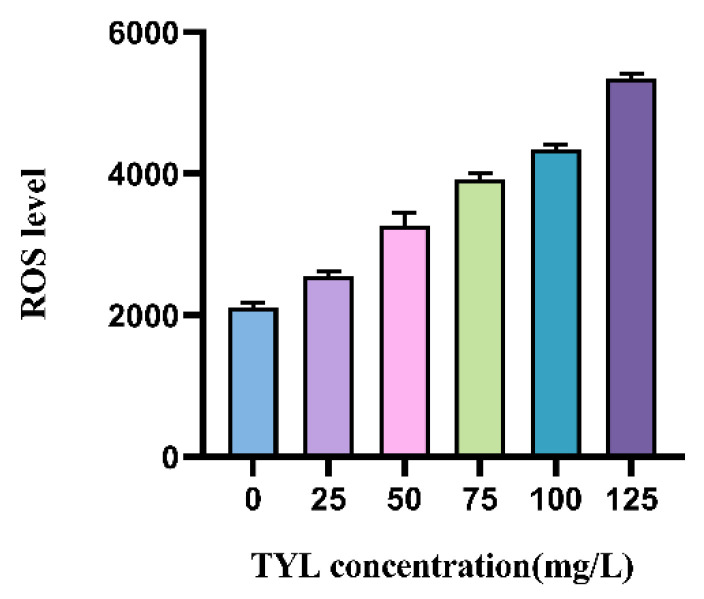
Effects of different concentrations of TYL exposure on ROS of strains.

Concentrations of ROS in strain TYL-A1 increased with increasing TYL concentrations (Figure 4), indicating that TYL stimulated the oxidation reaction of strain TYL-A1, and the reaction intensity was dose-dependent.

### 3.3. Antioxidant Reactions

**Figure 5:**
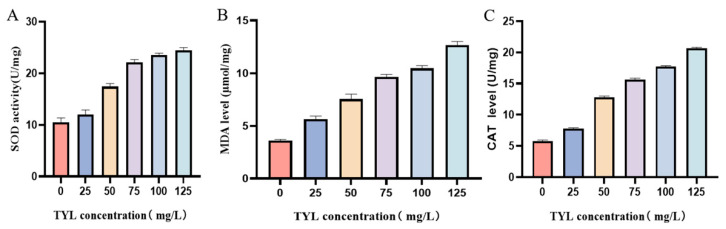
Effects of increasing concentrations of TYL on the content of: (**A**) SOD; (**B**) MDA; and (**C**) CAT in strain TYL-A1.

Three oxidation indices were assessed to explore different responses to various TYL stimuli. SOD is a key antioxidant enzyme that can protect cells from oxidative damage by catalyzing the disproportionation reaction of superoxide anion free radicals. With an increase in TYL concentration, SOD activity increased (Figure 5A), indicating that the strain scavenged oxygen free radicals. MDA is the final product of lipid peroxidation, and MDA content is an important index of antioxidant potential. As TYL concentration increased, MDA content also increased (Figure 5B), indicating that TYL induced oxidative damage. The primary job of the antioxidant enzyme CAT is to catalyze the breakdown of hydrogen peroxide (H_2_O_2_) into oxygen and water. With an increase in TYL concentration, CAT activity increased (Figure 5C).

### 3.4. ATP Content

**Figure 6:**
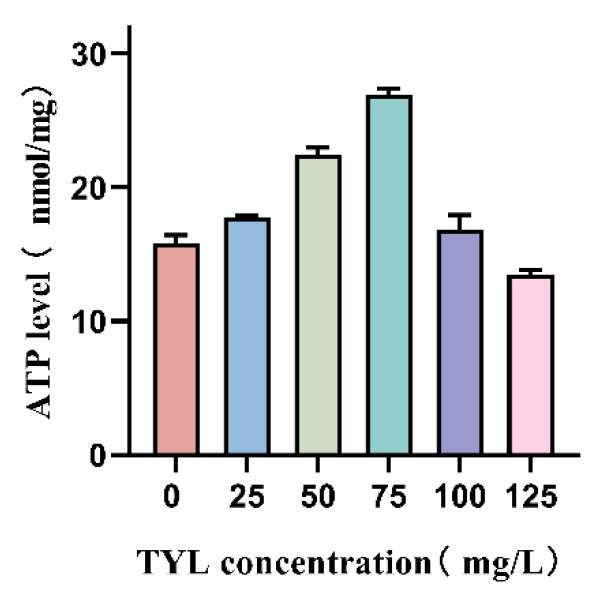
ATP content in strain TYL-A1 exposed to various concentrations of TYL.

In this study, as the TYL concentration increased, the trend for ATP content initially rose and then fell (Figure 6).

### 3.5. Transcriptome Sequencing Analysis

**Figure 7:**
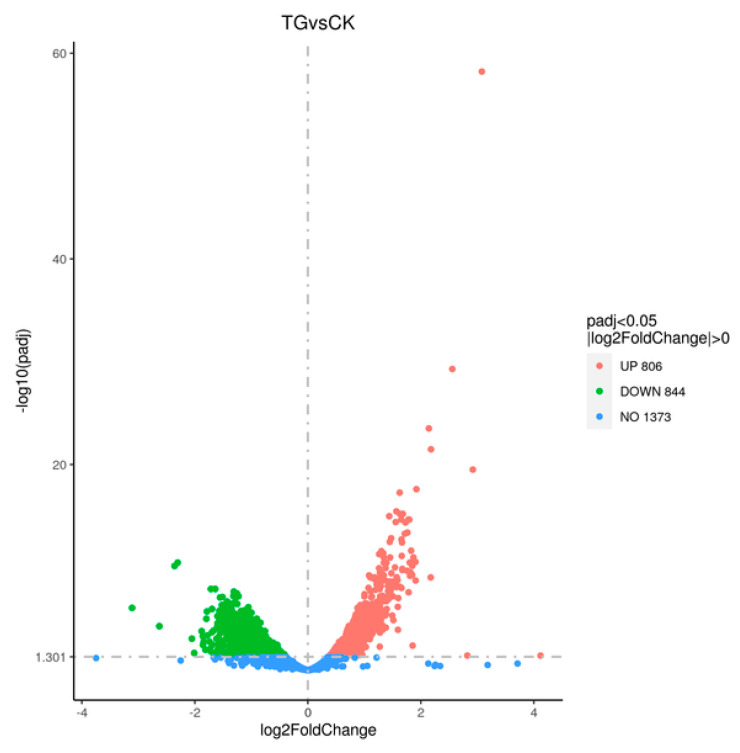
Volcano maps of all differentially expressed genes (DEGs) under tylosuin (TYL) stress in TYL-A1. Genes that are up- or down-regulated are indicated by red and green dots, respectively, and no differential gene expression is shown by blue dots.

The transcriptome sequencing of strain TYL-A1 was submitted to the GenBank database (Accession Number PRJNA1164894). Following TYL degradation, DEG between CK and TG were screened using GO and KEGG DEG analysis at a significance level of *p* < 0.05, |log2FC| ≥ 2. TYL influenced *K. gibsonii* TYL-A1 to produce 1650 DEGs. Among them, 806 genes were significantly up-regulated and 844 genes were significantly down-regulated (Figure 7).

#### 3.5.1. Metabolic Pathway of Strain TYL-A1 GO

**Figure 8:**
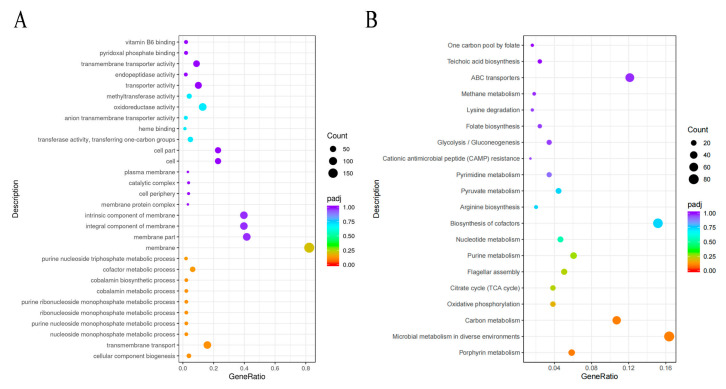
GO, KEGG enrichment analysis bubble diagram: (**A**) the top 30 enriched GO pathways and the number of DEGs under 75 mg/L TYL stress; and (**B**) the top 20 enriched KEGG pathways and the number of DEGs under 75 mg/L TYL stress.

Through GO enrichment analysis, pathways involved in DEG were identified. Gene functions were mainly divided into three types (Supplementary Table S1). Up-regulated genes were mainly distributed in molecular functions and biological processes, which were mainly biological processes completed by a variety of molecular activities. The most significantly enriched DEG in biological processes mainly included the following: cellular component biogenesis (GO:0044085), nucieoside monophosphate metabolism (GO:0009123), purine nucleoside monophosphate metabolism (GO:0009126), purine nucleoside triphosphate metabolism (GO:0009144), purine ribonucleoside monophosphate (GO:0009167), ribonucleoside monophosphate metabolism (GO:0009161), and RNA binding and structural molecular activity. Furthermore, DEGs most significantly enriched in molecular function were mainly endopeptidase activity (GO:0004175), oxidoreductase activity (GO:0016491), heme binding (GO:0020037), pyridoxal phosphate binding (GO:0030170), and vitamin B6 binding (GO:0070279). Considering the enrichment of DEG in MF, DEG is thought to have a direct bearing on TYL cell binding and secretory protein degradation. Tylosin degradation may occur as a result of this process, involving genes related to membrane transport in cellular components. TYL is integrated, and the related degrading protein components are released to break down the antibiotic. Cellular component refers to the location of the cell structure in which the gene product performs its function, which includes the membrane (GO:0016020), membrane part (GO:0044425), intrinsic component of membrane (GO:0016021), intrinsic component of membrane (GO:0031224), and cellular component (GO:0005575). These related genes are down-regulated and the other gene down-regulations are shown in Figure 8A.

#### 3.5.2. Metabolic Pathway of Strain TYL-A1 KEGG

Based on the KEGG analysis results, enrichment analysis was performed on 20 KEGG pathways with the highest levels of enrichment below 75 mg/L TYL stimulation (Figure 8B). The DEG of strain TYL-A1 was also rich in protein output, which may be related to the information transmission of strain TYL-T1. As shown in Supplementary Table S2, a total of 281 DEGs involved in 68 significant enrichment pathways were identified as significantly up-regulated, based on the KEGG analysis results. The main metabolic pathways were biosynthesis of secondary metabolites, microbial metabolism in diverse environments, biosynthesis of cofactors, ABC transporters, carbon metabolism, two-component system, biosynthesis of amino acids, purine metabolism, porphyrin metabolism, and quorum sensing. It is noteworthy that the biosynthesis of secondary metabolites was enriched to 139 DEGs. Of these, 98 there were 41 down-regulated DEGs and 41 were up-regulated DEGs. Furthermore, 55 DEGs were enriched by carbon metabolism, and more than half of the genes were up-regulated. These three processes had a key influence in the degradation of TYL.

### 3.6. Transcriptome Data Validation

**Figure 9:**
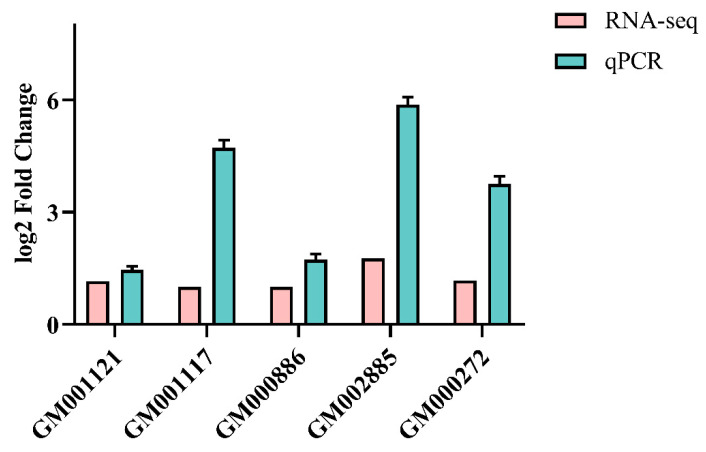
The gene expression of strain TYL-A1 under TYL exposure was verified by qPCR.

To validate the transcriptome data, five genes were chosen at random, and qPCR was used to determine each gene’s relative expression. The expression trend of the identified genes matched the transcriptome data and qPCR results (Figure 9 and Figure S2), demonstrating the validity and reproducibility of the transcriptome data.

### 3.7. Degradation of TYL by Possible Degradation Genes

**Figure 10:**
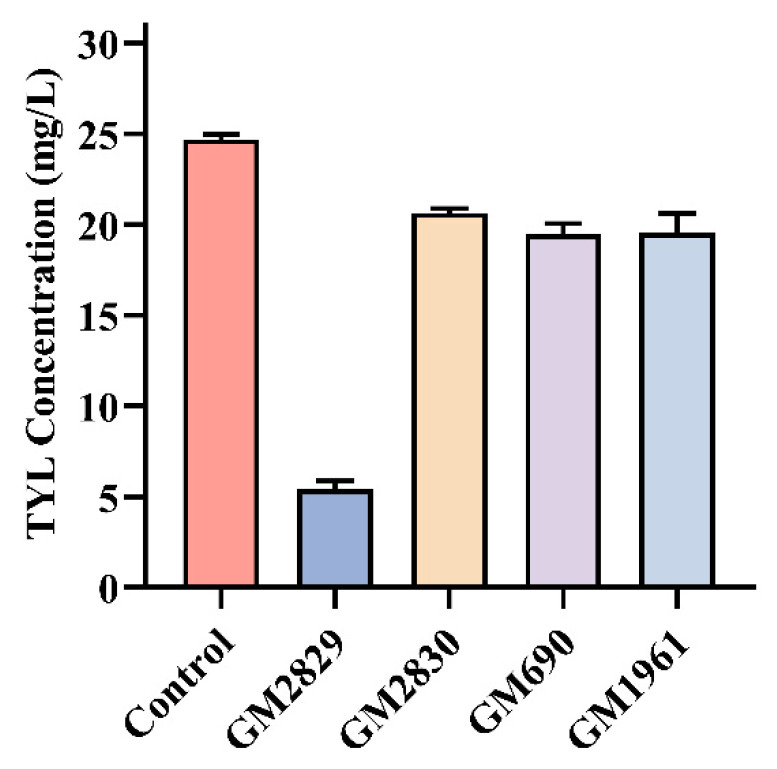
Degradation of TYL by recombinant bacteria.

Figure 10 shows the results of TYL degradation by heterologously expressed genes. Twenty-four hours after the culture, the GM002829 gene could significantly degrade TYL (*p* < 0.01), while the other four genes did not seem to have obvious degradation ability. It is suggested that GM002829 may be a key gene for the degradation of TYL.

## 4. Discussion

More and more attention has been paid to the serious environmental pollution caused by the wide application of TYL [25,26]. Among them, microbial degradation is widely used because of its safe and effective advantages [27,28]. The serious environmental pollution caused by the wide application of TYL has caused an increasing interest in recent studies. The preliminary intention of our research was to determine the effect of TYL on the strain TYL-A1 and to reveal the degradation mechanism of TYL-A1 based on the combined analysis of transcriptome data. Subsequently, the essential genes associated with degradation were identified to enable further studies on gene knockdown or cloned expression. Consequently, we decided to conduct the experiment using the TYL-efficient degrading bacteria TYL-A1. In this study, we explored the response mechanism of the strain under TYL exposure and then combined transcriptome sequencing to further explore the mechanism of interaction between them.

The bacterial cell membrane functions as a barrier with selective permeability, facilitating the transmission of information between the internal and external environments. This environment is conducive to a variety of biological processes within the cell [29]. The first part of the decomposition of organic pollutants by microorganisms first acts on the cell membrane. In our research, the cell membrane permeability of strain TYL-A1 was dose-dependent with the TYL concentration; with the increase in TYL concentration, the membrane permeability increased but this does not mean the cell membrane was destroyed. The increased permeability of strain TYL-A1 may be a regulatory mechanism of cell adaptation. There are research reports that increased membrane permeability during degradation improves degradation efficiency [30]. Zhao et al. found that the addition of Zn^2+^ increased the strain DNS10’s membrane permeability and promoted the degradation of atrazine in the strain [31]. Furthermore, the strain would degrade phenol more quickly if its membrane permeability increased [32]. In addition, microbial adsorption of organic matter is significantly influenced by cell surface hydrophobicity; an elevated CSH can improve the ability of microorganisms to adhere to pollutants, hastening the breakdown of pollutants [33]. This is also consistent with the results of this experiment. With the increase in TYL concentration, CSH first increased and then decreased, which could be the consequence of the elimination of extracellular hydrophobic compounds or TYL metabolites from the cell surface, which would lower CSH [34].

Under stable conditions, ROS concentrations in microorganisms will be in a balanced state; however, when microorganisms are in an adverse environment, ROS concentrations will increase. When ROS production surpasses the capacity of cells to eliminate them, the concentration increases and causes oxidative damage, affecting the development and metabolism of microbes [35]. In this experiment, ROS concentrations were dependent on TYL concentrations, and ROS concentrations increased with TYL concentrations, similar to a previous report that the ROS level in *Arthrobacter* sp. DNS10 increased with the increase in nicosulfuron concentration [36]. This is consistent with the results of TYL exposed cell membrane integrity was destroyed (Figure 1). Therefore, it can be concluded that TYL exposure damages strain TYL-A1’s cell membrane integrity by causing an excessive amount of ROS to be produced. In addition, the changes in SOD and CAT contents were also corresponding to the results of ROS and MDA contents.

As the main source of energy in cells, ATP is related to many mechanisms, such as cell necrosis and apoptosis, and has an important effect on cell function. Under environmental stress, bacteria need additional energy to maintain or restore their energy balance [37]. Cell necrosis and apoptosis are not synonymous. Several genes are activated, expressed, or regulated during the active process of apoptosis. It represents a positive response to promote adaptation to the living environment [38]. In the present study, as TYL concentration increased, ATP initially increased and then declined (Figure 6), indicating that TYL was toxic to the bacteria in a dose-dependent fashion. When bacteria are exposed to organic pollutants, bacteria often activate biosynthesis and metabolism of amino acids, resulting in amino acid deficiencies and up-regulation of amino acid synthesis genes [39]. Bacterial growth is also inhibited when amino acid concentrations in bacteria are out of balance.

Under TYL stress, strain TYL-A1 produced 1650 DEGs. Among them, 806 genes were significantly up-regulated and 844 DEGs were significantly down-regulated, indicating that microbial degradation of TYL was a complex process involving multiple genes. For example, *Klebsiella pneumoniae* TYL-T1 produced 262 DEGs during TYL degradation, with a substantial up-regulated of 164 DEGs and a significant down-regulated of 98 DEGs [19]. Additionally, it was reported that 1714 DEGs (1040 up-regulated and 674 down-regulated) were involved in the breakdown of BDE-47 by WZN-1 [40]. In the process of PET degradation by AIIW2, 1073 DEGs were up-regulated and 958 DEGs were down-regulated [41].

When degradation occurs, TYL is delivered in bacterial cells for metabolism. This process primarily involves genes related to energy anabolism, transmembrane transport, microbial transport, and redox pollutants. It is worth noting that in our study, these related genes were found to be enriched in the GO and KEGG databases, with most of them displaying up-regulated expression (Figures S1 and S2). According to research reports, the process by which organic contaminants degrade mainly occurs in cells, and microorganisms play a significant part in the transmembrane transport of organic pollutants [42]. The majority of DEGs were enriched and annotated in these pathways, which included carbon metabolism, microbial metabolism in various settings, cofactor biosynthesis, ABC transporters, and biosynthesis of secondary metabolites, according to KEGG pathway analysis. Among them, ABC transporters, are an important family of transmembrane transporters with a vital role in maintaining cell homeostasis and material transport through their unique structure and mechanism [43,44]. The breakdown of dimethyl phthalate involves ABC transporters, those transporters help cells absorb essential nutrients while also exporting toxic substances [45]. Therefore, the ABC transporter encoded by strain TYL-A1 had an important role in TYL degradation. In the Go-enriched pathway, DEGs have multiple terminology annotations in the three categories of molecular function, cell composition, and biological processes-membrane (Figure 2). Transmembrane transport, oxidoreductase activity, intrinsic component of the membrane, integral component of the membrane, membrane portion. Among the cell components, DEGs were significantly enriched, and most were down-regulated, indicating that TYL would affect growth.

The tricarboxylic acid cycle (TCA cycle) has a substantial role in cellular energy metabolism and substance conversion [46,47], and its complex and fine regulatory mechanism ensures the normal function of cells under many physiological conditions. The TCA cycle is typically enhanced when dangerous organic contaminants are catabolized [48]. Among the TCA-encoding genes in this study, only dihydrolipoic acid lysine residue succinyl transferase was down-regulated, whereas other genes were up-regulated, implying that TYL-A1 may use the TCA cycle to biodegrade TYL. Furthermore, the TCA cycle stimulated ROS production, inhibiting the growth of *Streptococcus epidermidis* [49], and was consistent with increased ROS concentrations in the current experiment. In addition, genes related to carbon metabolism and the oxidative phosphorylation pathway were up-regulated in this experiment, indicating responses to TYL stimulation to resist the oxidative damage caused by TYL stress. *Korshinsk peashrub* promoted carbon metabolism and oxidative phosphorylation of *Lentinus edodes* [50]. The expression of ribosome and carbon metabolism genes were up-regulated during the biodegradation of pyrene by fulvic acid [51]. Enhanced energy requirement under TYL stress was demonstrated by amplification of the energy metabolism pathway, and increasing ATP and NADH concentrations in response to oxidative stress [52].

In summary, this study elucidates the mechanism by which strain TYL-A1 degrades TYL. Our findings indicate that TYL degradation primarily occurs intracellularly, facilitated by membrane transport mechanisms during cellular uptake, and subsequently reliant on enzyme-catalyzed reactions within the bacterial cell. Transmembrane transport, oxidative stress and oxidoreductase activity genes are important genes affecting TYL degradation. TYL promoted the expression of oxidative phosphorylation, TCA cycle and carbon metabolism genes. Furthermore, there was an up-regulation in the expression of several possible degradative enzymes, including phosphotransferase and hydrolase. In addition, the degradation of TYL by the degradation enzyme gene GM002829 was also verified. Our study preliminarily determined the degradation of TYL by the GM002829 gene, but no further functional analysis was carried out. In the future, we will further study the degradation function of this degradation gene. These findings offer a theoretical foundation for additional research on TYL degradation.

## 5. Conclusions

The mechanism of response of strain TYL-A1 to TYL was studied. As the TYL concentration increased, there were increases in membrane permeability to promote TYL biodegradation. Furthermore, there were also increases in SOD, CAT and MDA, and ROS in the bacteria, indicating that the antioxidant reaction system was trying to mitigate cell damage. In total, 1650 DEGs were detected by transcriptome sequencing of strain TYL-A1, with 806 genes significantly up-regulated and 844 genes significantly down-regulated. TYL exposure induces the production of various degradative enzymes, including Phosphotransferase and glycoside hydrolase. During the degradation process, TYL metabolites accumulate within the cell, concurrently activating genes associated with the TCA cycle, oxidative phosphorylation, redox reactions and carbon metabolism. Furthermore, we validated the critical role of the GM002829 gene in TYL degradation. These results offer a theoretical basis for enhancing the biodegradation properties of TYL. Future studies will further investigate the functional roles of related genes to optimize TYL degradation efficiency.

## Data Availability

The original contributions presented in the study are included in the article/supplementary material, further inquiries can be directed to the corresponding authors.

## Supplementary Materials

The following supporting information can be downloaded at: https://www.mdpi.com/article/10.3390/microorganisms12122597/s1. Figure S1: GO keywords and integrated transcriptome assembly annotations and Figure S2: The gene expression of strain TYL-A1 under TYL exposure was verified by qPCR. Tables S1: DEGs enriched in GO database and Table S2: DEGs enriched by KEGG database.
