# Supplementary material for: Transcriptome of Kurthia gibsonii TYL-A1 Revealed the Biotransformation Mechanism of Tylosin
Source: Microorganisms. 2024 Dec 16;12(12):2597. doi: 10.3390/microorganisms12122597 (PMC11676290; doi:10.3390/microorganisms12122597)

Transcriptome of *Kurthia gibsonii* TYL-A1 Revealed the Biotransformation Mechanism of Tylosin

Ye Wang [1], Cuizhu Zhao [1], Boyu Zhao [1], Xinran Duan [1], Peng Hao [1], Xiaojun Liang [2], Lianyu Yang [1,*] and Yunhang Gao [1,*]

[1] College of Veterinary Medicine, Jilin Agricultural University, Changchun 130118, China; 18904404595@163.com (Y.W.); zhaocuizhu2024@163.com (C.Z.); zhaobooyu@163.com (B.Z.); 15048180317@163.com (X.D.); hhaopengg@163.com (P.H.)

[2] Institute of Animal Science, Ningxia Academy of Agriculture and Forestry Sciences, Yinchuan 750002, China; lxj0520@163.com

* Correspondence: yangly@jlau.edu.cn (L.Y.); gaoyunhang@163.com (Y.G.); Tel.: +86-13159752912 (Y.G.)

Figure S1 GO keywords and integrated transcriptome assembly annotations

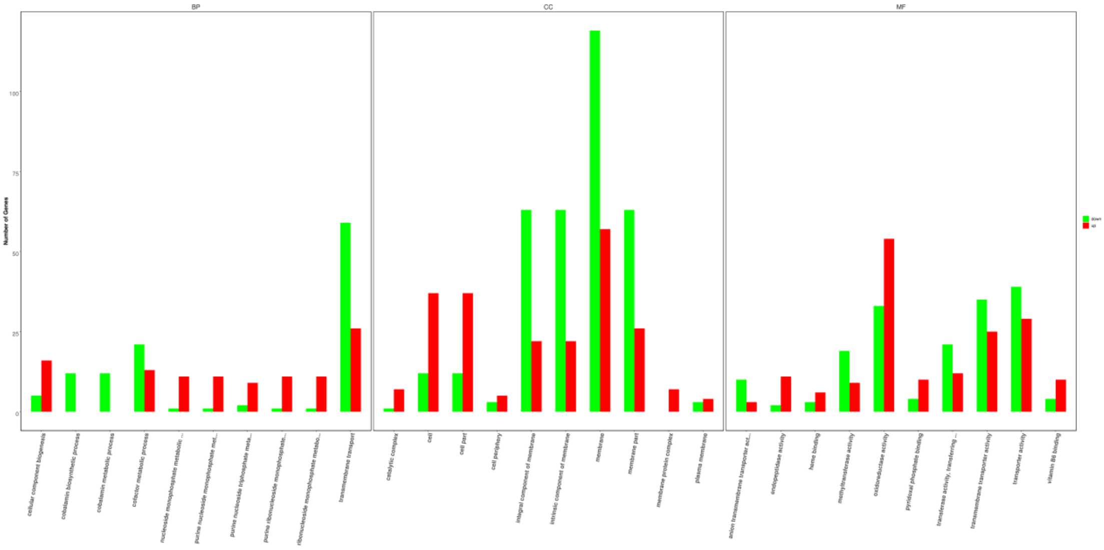

Table S1 DEGs enriched in GO database

Category	GOID	Description	GeneRatio	BgRatio	Up	Down
BP	GO:0044085	cellular component biogenesis	21/533	24/965	16	5
BP	GO:0055085	transmembrane transport	85/533	128/965	26	59
BP	GO:0009123	nucleoside monophosphate metabolic process	12/533	13/965	11	1
BP	GO:0009126	purine nucleoside monophosphate metabolic process	12/533	13/965	11	1
BP	GO:0009161	ribonucleoside monophosphate metabolic process	12/533	13/965	11	1
BP	GO:0009167	purine ribonucleoside monophosphate metabolic process	12/533	13/965	11	1
BP	GO:0009235	cobalamin metabolic process	12/533	13/965	0	12
BP	GO:0009236	cobalamin biosynthetic process	12/533	13/965	0	12
BP	GO:0051186	cofactor metabolic process	34/533	46/965	13	21
BP	GO:0009144	purine nucleoside triphosphate metabolic process	11/533	12/965	9	2
BP	GO:0009199	ribonucleoside triphosphate metabolic process	11/533	12/965	9	2
BP	GO:0009205	purine ribonucleoside triphosphate metabolic process	11/533	12/965	9	2
BP	GO:0022607	cellular component assembly	13/533	15/965	12	1

BP	GO:0009124	nucleoside monophosphate biosynthetic process	10/533	11/965	10	0
BP	GO:0009127	purine nucleoside monophosphate biosynthetic process	10/533	11/965	10	0
BP	GO:0009156	ribonucleoside monophosphate biosynthetic process	10/533	11/965	10	0
BP	GO:0009168	purine ribonucleoside monophosphate biosynthetic process	10/533	11/965	10	0
BP	GO:0046034	ATP metabolic process	10/533	11/965	9	1
BP	GO:0009141	nucleoside triphosphate metabolic process	12/533	14/965	9	3
BP	GO:0017144	drug metabolic process	28/533	39/965	15	13
BP	GO:0072521	purine-containing compound metabolic process	17/533	22/965	12	5
BP	GO:0009152	purine ribonucleotide biosynthetic process	11/533	13/965	10	1
BP	GO:0009260	ribonucleotide biosynthetic process	11/533	13/965	10	1
BP	GO:0046390	ribose phosphate biosynthetic process	11/533	13/965	10	1
BP	GO:0009150	purine ribonucleotide metabolic process	15/533	19/965	12	3
BP	GO:0009259	ribonucleotide metabolic process	15/533	19/965	12	3
BP	GO:0019693	ribose phosphate metabolic process	15/533	19/965	12	3
BP	GO:0033013	tetrapyrrole metabolic process	15/533	19/965	3	12
BP	GO:0033014	tetrapyrrole biosynthetic process	15/533	19/965	3	12
BP	GO:0072522	purine-containing compound biosynthetic process	13/533	16/965	10	3
BP	GO:0006163	purine nucleotide metabolic process	16/533	21/965	12	4
BP	GO:0071840	cellular component organization or biogenesis	31/533	45/965	23	8
BP	GO:0006164	purine nucleotide biosynthetic process	12/533	15/965	10	2
BP	GO:0090407	organophosphate biosynthetic process	24/533	34/965	16	8
BP	GO:0051179	localization	126/533	208/965	45	81

BP	GO:0006810	transport	119/533	196/965	41	78
BP	GO:0051234	establishment of localization	119/533	196/965	41	78
BP	GO:0051188	cofactor biosynthetic process	27/533	39/965	7	20
BP	GO:0055114	oxidation-reduction process	85/533	137/965	52	33
BP	GO:1901137	carbohydrate derivative biosynthetic process	11/533	14/965	10	1
BP	GO:1902600	proton transmembrane transport	9/533	11/965	8	1
BP	GO:0006793	phosphorus metabolic process	36/533	55/965	23	13
BP	GO:0006796	phosphate-containing compound metabolic process	34/533	52/965	21	13
BP	GO:0019637	organophosphate metabolic process	31/533	47/965	20	11
BP	GO:1901135	carbohydrate derivative metabolic process	31/533	47/965	23	8
BP	GO:0009165	nucleotide biosynthetic process	15/533	21/965	12	3
BP	GO:1901293	nucleoside phosphate biosynthetic process	15/533	21/965	12	3
BP	GO:0009117	nucleotide metabolic process	21/533	31/965	15	6
BP	GO:0006753	nucleoside phosphate metabolic process	22/533	33/965	15	7
BP	GO:0098655	cation transmembrane transport	9/533	12/965	8	1
BP	GO:0098660	inorganic ion transmembrane transport	9/533	12/965	8	1
BP	GO:0098662	inorganic cation transmembrane transport	9/533	12/965	8	1
BP	GO:0006766	vitamin metabolic process	15/533	22/965	3	12
BP	GO:0006767	water-soluble vitamin metabolic process	15/533	22/965	3	12
BP	GO:0009110	vitamin biosynthetic process	15/533	22/965	3	12
BP	GO:0042364	water-soluble vitamin biosynthetic process	15/533	22/965	3	12
BP	GO:0016043	cellular component organization	23/533	36/965	19	4

BP	GO:0006352	DNA-templated transcription, initiation	9/533	13/965	6	3
BP	GO:0006260	DNA replication	10/533	15/965	6	4
BP	GO:0006996	organelle organization	10/533	15/965	7	3
BP	GO:0034220	ion transmembrane transport	10/533	15/965	8	2
BP	GO:1901136	carbohydrate derivative catabolic process	7/533	10/965	3	4
BP	GO:0055086	nucleobase-containing small molecule metabolic process	29/533	48/965	20	9
BP	GO:0006820	anion transport	14/533	22/965	3	11
BP	GO:0006811	ion transport	35/533	60/965	17	18
BP	GO:0005975	carbohydrate metabolic process	18/533	30/965	14	4
BP	GO:0034470	ncRNA processing	10/533	16/965	6	4
BP	GO:0006812	cation transport	23/533	39/965	14	9
BP	GO:0015672	monovalent inorganic cation transport	11/533	18/965	8	3
BP	GO:0015074	DNA integration	7/533	11/965	1	6
BP	GO:0042592	homeostatic process	7/533	11/965	6	1
BP	GO:1901565	organonitrogen compound catabolic process	7/533	11/965	4	3
BP	GO:0006396	RNA processing	16/533	27/965	9	7
BP	GO:0006732	coenzyme metabolic process	16/533	27/965	7	9
BP	GO:0034660	ncRNA metabolic process	21/533	36/965	11	10
BP	GO:0044255	cellular lipid metabolic process	12/533	20/965	10	2
BP	GO:0006790	sulfur compound metabolic process	8/533	13/965	7	1
BP	GO:0006928	movement of cell or subcellular component	8/533	13/965	5	3
BP	GO:0015711	organic anion transport	9/533	15/965	1	8

BP	GO:0015849	organic acid transport	9/533	15/965	1	8
BP	GO:0046942	carboxylic acid transport	9/533	15/965	1	8
BP	GO:0044281	small molecule metabolic process	85/533	152/965	49	36
BP	GO:0018130	heterocycle biosynthetic process	111/533	199/965	59	52
BP	GO:0006629	lipid metabolic process	16/533	28/965	12	4
BP	GO:0006418	tRNA aminoacylation for protein translation	11/533	19/965	5	6
BP	GO:0044248	cellular catabolic process	11/533	19/965	6	5
BP	GO:0009057	macromolecule catabolic process	6/533	10/965	3	3
BP	GO:0043933	protein-containing complex subunit organization	6/533	10/965	5	1
BP	GO:0051276	chromosome organization	6/533	10/965	4	2
BP	GO:0006508	proteolysis	18/533	32/965	12	6
BP	GO:0001539	cilium or flagellum-dependent cell motility	7/533	12/965	4	3
BP	GO:0048870	cell motility	7/533	12/965	4	3
BP	GO:0051674	localization of cell	7/533	12/965	4	3
BP	GO:0071973	bacterial-type flagellum-dependent cell motility	7/533	12/965	4	3
BP	GO:0097588	archaeal or bacterial-type flagellum-dependent cell motility	7/533	12/965	4	3
BP	GO:0008610	lipid biosynthetic process	8/533	14/965	8	0
BP	GO:1901362	organic cyclic compound biosynthetic process	111/533	202/965	59	52
BP	GO:0008104	protein localization	9/533	16/965	5	4
BP	GO:0015031	protein transport	9/533	16/965	5	4
BP	GO:0033036	macromolecule localization	9/533	16/965	5	4
BP	GO:0045184	establishment of protein localization	9/533	16/965	5	4

BP	GO:0040011	locomotion	10/533	18/965	7	3
BP	GO:0006310	DNA recombination	11/533	20/965	5	6
BP	GO:0043038	amino acid activation	11/533	20/965	5	6
BP	GO:0043039	tRNA aminoacylation	11/533	20/965	5	6
BP	GO:0009056	catabolic process	13/533	24/965	8	5
BP	GO:1901575	organic substance catabolic process	13/533	24/965	8	5
BP	GO:0019438	aromatic compound biosynthetic process	104/533	191/965	56	48
BP	GO:0006865	amino acid transport	6/533	11/965	1	5
BP	GO:0034404	nucleobase-containing small molecule biosynthetic process	6/533	11/965	4	2
BP	GO:0046483	heterocycle metabolic process	190/533	348/965	100	90
BP	GO:0042221	response to chemical	8/533	15/965	4	4
BP	GO:0009116	nucleoside metabolic process	9/533	17/965	7	2
BP	GO:1901657	glycosyl compound metabolic process	9/533	17/965	7	2
BP	GO:0006259	DNA metabolic process	39/533	73/965	20	19
BP	GO:0034654	nucleobase-containing compound biosynthetic process	84/533	156/965	53	31
BP	GO:0009108	coenzyme biosynthetic process	12/533	23/965	5	7
BP	GO:1901360	organic cyclic compound metabolic process	190/533	351/965	100	90
BP	GO:0046434	organophosphate catabolic process	5/533	10/965	3	2
BP	GO:0019439	aromatic compound catabolic process	6/533	12/965	4	2
BP	GO:0044270	cellular nitrogen compound catabolic process	6/533	12/965	4	2
BP	GO:0046700	heterocycle catabolic process	6/533	12/965	4	2
BP	GO:1901361	organic cyclic compound catabolic process	6/533	12/965	4	2

BP	GO:0006725	cellular aromatic compound metabolic process	185/533	343/965	97	88
BP	GO:0071705	nitrogen compound transport	20/533	39/965	7	13
BP	GO:0071702	organic substance transport	23/533	45/965	7	16
BP	GO:0015833	peptide transport	12/533	24/965	5	7
BP	GO:0006139	nucleobase-containing compound metabolic process	162/533	302/965	93	69
BP	GO:0042886	amide transport	13/533	26/965	5	8
BP	GO:0006399	tRNA metabolic process	14/533	28/965	8	6
BP	GO:0065008	regulation of biological quality	8/533	17/965	7	1
BP	GO:0032787	monocarboxylic acid metabolic process	7/533	15/965	5	2
BP	GO:0043412	macromolecule modification	14/533	29/965	7	7
BP	GO:0034655	nucleobase-containing compound catabolic process	5/533	11/965	3	2
BP	GO:0006351	transcription, DNA-templated	66/533	128/965	40	26
BP	GO:0032774	RNA biosynthetic process	66/533	128/965	40	26
BP	GO:0097659	nucleic acid-templated transcription	66/533	128/965	40	26
BP	GO:0019219	regulation of nucleobase-containing compound metabolic process	63/533	123/965	38	25
BP	GO:0031323	regulation of cellular metabolic process	63/533	123/965	38	25
BP	GO:0051171	regulation of nitrogen compound metabolic process	63/533	123/965	38	25
BP	GO:0060255	regulation of macromolecule metabolic process	64/533	125/965	38	26
BP	GO:0090304	nucleic acid metabolic process	132/533	252/965	73	59
BP	GO:0009889	regulation of biosynthetic process	62/533	122/965	37	25
BP	GO:0010556	regulation of macromolecule biosynthetic process	62/533	122/965	37	25
BP	GO:00313	regulation of cellular	62/533	122/	37	25

	26	biosynthetic process		965		
BP	GO:2000112	regulation of cellular macromolecule biosynthetic process	62/533	122/965	37	25
BP	GO:0016070	RNA metabolic process	94/533	182/965	54	40
BP	GO:0030001	metal ion transport	8/533	18/965	5	3
BP	GO:0019222	regulation of metabolic process	64/533	126/965	38	26
BP	GO:0050789	regulation of biological process	95/533	184/965	53	42
BP	GO:0006355	regulation of transcription, DNA-templated	61/533	121/965	37	24
BP	GO:0051252	regulation of RNA metabolic process	61/533	121/965	37	24
BP	GO:1903506	regulation of nucleic acid-templated transcription	61/533	121/965	37	24
BP	GO:2001141	regulation of RNA biosynthetic process	61/533	121/965	37	24
BP	GO:0050794	regulation of cellular process	93/533	181/965	52	41
BP	GO:0009451	RNA modification	5/533	12/965	2	3
BP	GO:0010468	regulation of gene expression	62/533	123/965	37	25
BP	GO:0080090	regulation of primary metabolic process	63/533	125/965	38	25
BP	GO:0044283	small molecule biosynthetic process	32/533	66/965	14	18
BP	GO:0000160	phosphorelay signal transduction system	19/533	41/965	10	9
BP	GO:0035556	intracellular signal transduction	19/533	41/965	10	9
BP	GO:0065007	biological regulation	97/533	191/965	54	43
BP	GO:0006082	organic acid metabolic process	38/533	80/965	23	15
BP	GO:0019752	carboxylic acid metabolic process	37/533	79/965	23	14
BP	GO:0043436	oxoacid metabolic process	37/533	79/965	23	14
BP	GO:0007165	signal transduction	32/533	70/965	14	18

BP	GO:0023052	signaling	32/533	70/965	14	18
BP	GO:0007154	cell communication	32/533	71/965	14	18
BP	GO:0044271	cellular nitrogen compound biosynthetic process	127/533	257/965	73	54
BP	GO:1901564	organonitrogen compound metabolic process	112/533	230/965	66	46
BP	GO:0044237	cellular metabolic process	250/533	485/965	149	101
BP	GO:0044260	cellular macromolecule metabolic process	139/533	283/965	78	61
BP	GO:0006520	cellular amino acid metabolic process	22/533	56/965	14	8
BP	GO:1901576	organic substance biosynthetic process	159/533	322/965	94	65
BP	GO:0009058	biosynthetic process	174/533	350/965	105	69
BP	GO:0043170	macromolecule metabolic process	176/533	354/965	100	76
BP	GO:0034641	cellular nitrogen compound metabolic process	196/533	392/965	109	87
BP	GO:0044249	cellular biosynthetic process	156/533	318/965	92	64
BP	GO:0019538	protein metabolic process	48/533	111/965	30	18
BP	GO:0010467	gene expression	104/533	221/965	63	41
BP	GO:0034645	cellular macromolecule biosynthetic process	97/533	208/965	60	37
BP	GO:0009059	macromolecule biosynthetic process	98/533	210/965	60	38
BP	GO:1901566	organonitrogen compound biosynthetic process	66/533	149/965	34	32
BP	GO:0051716	cellular response to stimulus	36/533	90/965	17	19
BP	GO:0044267	cellular protein metabolic process	30/533	78/965	18	12
BP	GO:0016053	organic acid biosynthetic process	10/533	34/965	6	4
BP	GO:0046394	carboxylic acid biosynthetic process	10/533	34/965	6	4
BP	GO:0006807	nitrogen compound metabolic process	237/533	474/965	135	102

BP	GO:0006281	DNA repair	4/533	19/965	3	1
BP	GO:0006974	cellular response to DNA damage stimulus	4/533	19/965	3	1
BP	GO:0033554	cellular response to stress	4/533	19/965	3	1
BP	GO:1901607	alpha-amino acid biosynthetic process	4/533	19/965	2	2
BP	GO:0044238	primary metabolic process	240/533	482/965	150	90
BP	GO:0050896	response to stimulus	41/533	104/965	18	23
BP	GO:0008652	cellular amino acid biosynthetic process	5/533	23/965	3	2
BP	GO:1901605	alpha-amino acid metabolic process	6/533	27/965	4	2
BP	GO:0006412	translation	21/533	64/965	14	7
BP	GO:0006518	peptide metabolic process	21/533	64/965	14	7
BP	GO:0043043	peptide biosynthetic process	21/533	64/965	14	7
BP	GO:0043603	cellular amide metabolic process	24/533	71/965	14	10
BP	GO:0006950	response to stress	4/533	23/965	3	1
BP	GO:0043604	amide biosynthetic process	23/533	70/965	14	9
CC	GO:0016020	membrane	176/214	294/379	57	119
CC	GO:0044425	membrane part	89/214	146/379	26	63
CC	GO:0016021	integral component of membrane	85/214	142/379	22	63
CC	GO:0031224	intrinsic component of membrane	85/214	142/379	22	63
CC	GO:0098796	membrane protein complex	7/214	10/379	7	0
CC	GO:0071944	cell periphery	8/214	14/379	5	3
CC	GO:1902494	catalytic complex	8/214	14/379	7	1
CC	GO:0005886	plasma membrane	7/214	13/379	4	3

CC	GO:0005623	cell	49/214	101/379	37	12
CC	GO:0044464	cell part	49/214	101/379	37	12
CC	GO:0005622	intracellular	36/214	81/379	31	5
CC	GO:0044424	intracellular part	32/214	75/379	27	5
CC	GO:0005737	cytoplasm	23/214	62/379	19	4
CC	GO:0032991	protein-containing complex	22/214	61/379	19	3
CC	GO:0043226	organelle	15/214	49/379	11	4
CC	GO:0043228	non-membrane-bounded organelle	15/214	49/379	11	4
CC	GO:0044444	cytoplasmic part	11/214	43/379	10	1
CC	GO:0043229	intracellular organelle	11/214	44/379	10	1
CC	GO:0043232	intracellular non-membrane-bounded organelle	11/214	44/379	10	1
CC	GO:1990904	ribonucleoprotein complex	9/214	41/379	8	1
CC	GO:0005840	ribosome	8/214	40/379	7	1
CC	GO:0005575	cellular_component	214/214	379/379	86	128
MF	GO:0016741	transferase activity, transferring one-carbon groups	33/674	46/1243	12	21
MF	GO:0020037	heme binding	9/674	10/1243	6	3
MF	GO:0008509	anion transmembrane transporter activity	13/674	16/1243	3	10
MF	GO:0016491	oxidoreductase activity	87/674	140/1243	54	33
MF	GO:0008168	methyltransferase activity	28/674	40/1243	9	19
MF	GO:0005215	transporter activity	68/674	110/1243	29	39
MF	GO:0004175	endopeptidase activity	13/674	19/1243	11	2
MF	GO:0022857	transmembrane transporter activity	60/674	101/1243	25	35

MF	GO:0030170	pyridoxal phosphate binding	14/674	21/1243	10	4
MF	GO:0070279	vitamin B6 binding	14/674	21/1243	10	4
MF	GO:0005342	organic acid transmembrane transporter activity	8/674	11/1243	1	7
MF	GO:0008514	organic anion transmembrane transporter activity	8/674	11/1243	1	7
MF	GO:0046943	carboxylic acid transmembrane transporter activity	8/674	11/1243	1	7
MF	GO:0042626	ATPase activity, coupled to transmembrane movement of substances	11/674	16/1243	6	5
MF	GO:0043492	ATPase activity, coupled to movement of substances	11/674	16/1243	6	5
MF	GO:0046906	tetrapyrrole binding	11/674	16/1243	8	3
MF	GO:0048037	cofactor binding	57/674	98/1243	37	20
MF	GO:0015399	primary active transmembrane transporter activity	11/674	17/1243	6	5
MF	GO:0015405	P-P-bond-hydrolysis-driven transmembrane transporter activity	11/674	17/1243	6	5
MF	GO:0070011	peptidase activity, acting on L-amino acid peptides	19/674	31/1243	13	6
MF	GO:0042623	ATPase activity, coupled	15/674	24/1243	8	7
MF	GO:0016830	carbon-carbon lyase activity	8/674	12/1243	5	3
MF	GO:0001882	nucleoside binding	16/674	26/1243	10	6
MF	GO:0001883	purine nucleoside binding	16/674	26/1243	10	6
MF	GO:0005525	GTP binding	16/674	26/1243	10	6
MF	GO:0032549	ribonucleoside binding	16/674	26/1243	10	6
MF	GO:0032550	purine ribonucleoside binding	16/674	26/1243	10	6
MF	GO:0050662	coenzyme binding	35/674	60/1243	22	13
MF	GO:0140097	catalytic activity, acting on DNA	18/674	30/1243	11	7
MF	GO:00082	peptidase activity	21/674	36/1	14	7

	33			243		
MF	GO:0016740	transferase activity	99/674	178/1243	51	48
MF	GO:0015078	proton transmembrane transporter activity	7/674	11/1243	6	1
MF	GO:0043167	ion binding	179/674	325/1243	98	81
MF	GO:0015318	inorganic molecular entity transmembrane transporter activity	22/674	38/1243	10	12
MF	GO:0043169	cation binding	33/674	58/1243	26	7
MF	GO:0035639	purine ribonucleoside triphosphate binding	120/674	217/1243	57	63
MF	GO:0003700	DNA binding transcription factor activity	39/674	69/1243	23	16
MF	GO:0140110	transcription regulator activity	39/674	69/1243	23	16
MF	GO:0043168	anion binding	151/674	274/1243	76	75
MF	GO:0019842	vitamin binding	18/674	31/1243	13	5
MF	GO:0032553	ribonucleotide binding	122/674	221/1243	58	64
MF	GO:0046872	metal ion binding	31/674	55/1243	24	7
MF	GO:0097367	carbohydrate derivative binding	123/674	224/1243	59	64
MF	GO:0019001	guanyl nucleotide binding	16/674	28/1243	10	6
MF	GO:0032561	guanyl ribonucleotide binding	16/674	28/1243	10	6
MF	GO:0032555	purine ribonucleotide binding	120/674	219/1243	57	63
MF	GO:0042578	phosphoric ester hydrolase activity	6/674	10/1243	3	3
MF	GO:0017076	purine nucleotide binding	120/674	220/1243	57	63
MF	GO:0016853	isomerase activity	19/674	34/1243	12	7
MF	GO:0036094	small molecule binding	160/674	294/1243	83	77
MF	GO:0005524	ATP binding	104/674	191/1243	47	57

MF	GO:0032559	adenyl ribonucleotide binding	104/674	191/1243	47	57
MF	GO:0016780	phosphotransferase activity, for other substituted phosphate groups	7/674	12/1243	2	5
MF	GO:0016879	ligase activity, forming carbon-nitrogen bonds	7/674	12/1243	4	3
MF	GO:0015075	ion transmembrane transporter activity	27/674	49/1243	11	16
MF	GO:0000166	nucleotide binding	138/674	254/1243	66	72
MF	GO:1901265	nucleoside phosphate binding	138/674	254/1243	66	72
MF	GO:0140096	catalytic activity, acting on a protein	36/674	66/1243	22	14
MF	GO:0008144	drug binding	123/674	227/1243	61	62
MF	GO:0030554	adenyl nucleotide binding	104/674	192/1243	47	57
MF	GO:0016829	lyase activity	17/674	31/1243	11	6
MF	GO:0140098	catalytic activity, acting on RNA	25/674	46/1243	14	11
MF	GO:0046914	transition metal ion binding	11/674	20/1243	10	1
MF	GO:0016772	transferase activity, transferring phosphorus-containing groups	45/674	84/1243	24	21
MF	GO:0016874	ligase activity	23/674	43/1243	11	12
MF	GO:0003924	GTPase activity	6/674	11/1243	3	3
MF	GO:0008236	serine-type peptidase activity	9/674	17/1243	5	4
MF	GO:0017171	serine hydrolase activity	9/674	17/1243	5	4
MF	GO:0015077	monovalent inorganic cation transmembrane transporter activity	10/674	19/1243	6	4
MF	GO:0016779	nucleotidyltransferase activity	10/674	19/1243	8	2
MF	GO:0004812	aminoacyl-tRNA ligase activity	11/674	21/1243	5	6
MF	GO:0016875	ligase activity, forming carbon-oxygen bonds	11/674	21/1243	5	6
MF	GO:0051536	iron-sulfur cluster binding	12/674	23/1243	6	6

MF	GO:0051540	metal cluster binding	12/674	23/1243	6	6
MF	GO:0022804	active transmembrane transporter activity	20/674	39/1243	8	12
MF	GO:0004252	serine-type endopeptidase activity	5/674	10/1243	4	1
MF	GO:0016866	intramolecular transferase activity	7/674	14/1243	4	3
MF	GO:0016614	oxidoreductase activity, acting on CH-OH group of donors	10/674	20/1243	6	4
MF	GO:0022890	inorganic cation transmembrane transporter activity	12/674	24/1243	7	5
MF	GO:0016887	ATPase activity	49/674	95/1243	17	32
MF	GO:0140101	catalytic activity, acting on a tRNA	15/674	30/1243	8	7
MF	GO:0016810	hydrolase activity, acting on carbon-nitrogen (but not peptide) bonds	10/674	21/1243	7	3
MF	GO:0050660	flavin adenine dinucleotide binding	9/674	19/1243	3	6
MF	GO:0008324	cation transmembrane transporter activity	17/674	35/1243	8	9
MF	GO:0016627	oxidoreductase activity, acting on the CH-CH group of donors	6/674	13/1243	2	4
MF	GO:0016301	kinase activity	21/674	43/1243	10	11
MF	GO:0008270	zinc ion binding	5/674	11/1243	5	0
MF	GO:0015293	symporter activity	5/674	11/1243	1	4
MF	GO:0017111	nucleoside-triphosphatase activity	56/674	111/1243	20	36
MF	GO:0016462	pyrophosphatase activity	58/674	115/1243	21	37
MF	GO:0016817	hydrolase activity, acting on acid anhydrides	58/674	115/1243	21	37
MF	GO:0016818	hydrolase activity, acting on acid anhydrides, in phosphorus-containing anhydrides	58/674	115/1243	21	37
MF	GO:0005515	protein binding	23/674	48/1243	16	7
MF	GO:0016746	transferase activity, transferring acyl groups	9/674	20/1243	7	2

MF	GO:0016616	oxidoreductase activity, acting on the CH-OH group of donors, NAD or NADP as acceptor	8/674	18/1243	5	3
MF	GO:0016835	carbon-oxygen lyase activity	4/674	10/1243	3	1
MF	GO:1901681	sulfur compound binding	4/674	10/1243	2	2
MF	GO:0043565	sequence-specific DNA binding	12/674	27/1243	7	5
MF	GO:0004672	protein kinase activity	11/674	25/1243	4	7
MF	GO:0004871	signal transducer activity	11/674	25/1243	5	6
MF	GO:0016787	hydrolase activity	135/674	265/1243	64	71
MF	GO:0016773	phosphotransferase activity, alcohol group as acceptor	18/674	40/1243	7	11
MF	GO:0003677	DNA binding	86/674	173/1243	52	34
MF	GO:0046873	metal ion transmembrane transporter activity	5/674	13/1243	1	4
MF	GO:0000155	phosphorelay sensor kinase activity	10/674	24/1243	4	6
MF	GO:0004673	protein histidine kinase activity	10/674	24/1243	4	6
MF	GO:0016775	phosphotransferase activity, nitrogenous group as acceptor	10/674	24/1243	4	6
MF	GO:0038023	signaling receptor activity	10/674	24/1243	4	6
MF	GO:0060089	molecular transducer activity	10/674	24/1243	4	6
MF	GO:0016747	transferase activity, transferring acyl groups other than amino-acyl groups	4/674	11/1243	3	1
MF	GO:0016765	transferase activity, transferring alkyl or aryl (other than methyl) groups	4/674	11/1243	2	2
MF	GO:0016811	hydrolase activity, acting on carbon-nitrogen (but not peptide) bonds, in linear amides	4/674	11/1243	3	1
MF	GO:0015291	secondary active transmembrane transporter activity	7/674	18/1243	1	6
MF	GO:0016788	hydrolase activity, acting on ester bonds	17/674	40/1243	9	8

MF	GO:0003723	RNA binding	24/674	55/1243	13	11
MF	GO:0004519	endonuclease activity	3/674	10/1243	1	2
MF	GO:0004518	nuclease activity	5/674	16/1243	2	3
MF	GO:0003676	nucleic acid binding	127/674	266/1243	74	53
MF	GO:0046983	protein dimerization activity	2/674	10/1243	2	0
MF	GO:0005198	structural molecule activity	12/674	44/1243	10	2
MF	GO:0003735	structural constituent of ribosome	9/674	40/1243	7	2

Table S2 DEGs enriched by KEGG database

KEGGID	Description	GeneRatio	BgRatio	Up	Down
kur00860	Porphyrin metabolism	29/495	38/958	6	23
kur01120	Microbial metabolism in diverse environments	81/495	129/958	53	28
kur01200	Carbon metabolism	53/495	80/958	47	6
kur00190	Oxidative phosphorylation	19/495	25/958	18	1
kur00020	Citrate cycle (TCA cycle)	19/495	26/958	18	1
kur02040	Flagellar assembly	25/495	36/958	12	13
kur00230	Purine metabolism	30/495	45/958	17	13
kur01232	Nucleotide metabolism	23/495	35/958	17	6
kur01240	Biosynthesis of cofactors	75/495	131/958	33	42
kur00220	Arginine biosynthesis	10/495	14/958	9	1
kur00620	Pyruvate metabolism	22/495	35/958	18	4
kur00240	Pyrimidine metabolism	17/495	27/958	14	3
kur01503	Cationic antimicrobial peptide (CAMP) resistance	7/495	10/958	6	1
kur00010	Glycolysis / Gluconeogenesis	17/495	28/958	13	4
kur00790	Folate biosynthesis	12/495	19/958	1	11
kur00310	Lysine degradation	8/495	12/958	2	6
kur00680	Methane metabolism	9/495	14/958	7	2
kur02010	ABC transporters	60/495	109/958	22	38
kur00552	Teichoic acid biosynthesis	12/495	20/958	8	4
kur00670	One carbon pool by folate	8/495	13/958	4	4
kur00640	Propanoate metabolism	15/495	26/958	10	5
kur00250	Alanine, aspartate and glutamate metabolism	16/495	28/958	11	5
kur00630	Glyoxylate and dicarboxylate	16/495	28/958	12	4

	metabolism				
kur03030	DNA replication	10/495	17/958	8	2
kur00920	Sulfur metabolism	11/495	19/958	3	8
kur02030	Bacterial chemotaxis	16/495	29/958	6	10
kur03060	Protein export	8/495	14/958	8	0
kur03018	RNA degradation	9/495	16/958	5	4
kur00650	Butanoate metabolism	12/495	22/958	6	6
kur02024	Quorum sensing	26/495	49/958	10	16
kur00450	Selenocompound metabolism	6/495	11/958	5	1
kur00561	Glycerolipid metabolism	6/495	11/958	4	2
kur03070	Bacterial secretion system	6/495	11/958	6	0
kur01110	Biosynthesis of secondary metabolites	139/495	270/958	98	41
kur00550	Peptidoglycan biosynthesis	12/495	23/958	6	6
kur00260	Glycine, serine and threonine metabolism	14/495	28/958	8	6
kur03430	Mismatch repair	10/495	20/958	5	5
kur00300	Lysine biosynthesis	8/495	16/958	4	4
kur00900	Terpenoid backbone biosynthesis	6/495	12/958	6	0
kur00970	Aminoacyl-tRNA biosynthesis	12/495	25/958	6	6
kur03440	Homologous recombination	10/495	21/958	6	4
kur00030	Pentose phosphate pathway	5/495	11/958	5	0
kur00740	Riboflavin metabolism	5/495	11/958	5	0
kur00280	Valine, leucine and isoleucine degradation	9/495	20/958	5	4
kur01210	2-Oxocarboxylic acid metabolism	9/495	20/958	8	1
kur00330	Arginine and proline metabolism	8/495	18/958	3	5
kur00470	D-Amino acid metabolism	6/495	14/958	3	3
kur04122	Sulfur relay system	6/495	14/958	2	4
kur00071	Fatty acid degradation	5/495	12/958	3	2
kur01501	beta-Lactam resistance	5/495	12/958	2	3
kur00564	Glycerophospholipid metabolism	9/495	21/958	6	3
kur03410	Base excision repair	4/495	10/958	2	2
kur00730	Thiamine metabolism	7/495	17/958	2	5
kur02020	Two-component system	47/495	101/958	24	23
kur00270	Cysteine and methionine metabolism	13/495	31/958	10	3
kur00760	Nicotinate and nicotinamide metabolism	4/495	11/958	3	1
kur00780	Biotin metabolism	4/495	11/958	2	2
kur00061	Fatty acid biosynthesis	7/495	18/958	5	2
kur00770	Pantothenate and CoA biosynthesis	6/495	17/958	1	5

kur00362	Benzoate degradation	3/495	10/958	1	2
kur01250	Biosynthesis of nucleotide sugars	3/495	10/958	3	0
kur00340	Histidine metabolism	4/495	14/958	1	3
kur00520	Amino sugar and nucleotide sugar metabolism	4/495	14/958	4	0
kur01212	Fatty acid metabolism	8/495	24/958	5	3
kur01230	Biosynthesis of amino acids	44/495	108/958	34	10
kur00400	Phenylalanine, tyrosine and tryptophan biosynthesis	4/495	19/958	3	1
kur00290	Valine, leucine and isoleucine biosynthesis	1/495	10/958	0	1
kur03010	Ribosome	14/495	51/958	11	3

Figure S2 The gene expression of strain TYL-A1 under TYL exposure was verified by qPCR.

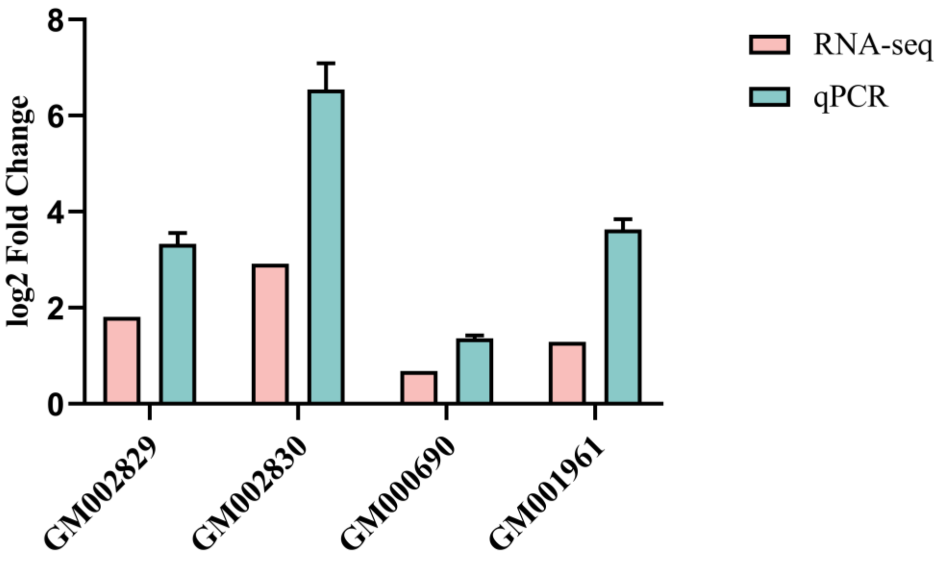

Supplement: Supplementary file 1 [file microorganisms-12-02597-s001.zip › microorganisms-3324762-supplementary.pdf]
